# Functional foods of sub‐Saharan Africa and their implications in the management of type 2 diabetes: A review

**DOI:** 10.1002/fsn3.3764

**Published:** 2023-10-16

**Authors:** Rosane Matsinkou Soh, Wilfred Ngaha Damndja, Nicolas Njintang Yanou

**Affiliations:** ^1^ Department of Food Science and Nutrition, National School of Agro‐Industrial Sciences University of Ngaoundere Ngaoundere Cameroon; ^2^ Faculty of Sciences University of Ngaoundere Ngaoundere Cameroon

**Keywords:** bioactive compounds, functional foods, management, sub‐Saharan Africa, type 2 diabetes

## Abstract

Throughout the world, the prevalence of metabolic diseases in general and type 2 diabetes (T2DM) in particular is constantly growing, and sub‐Saharan Africa (SSA) is not spared. The use of functional foods is a more practical option among the different approaches used in the management of T2DM owing to the fact that they are relatively less costly, safer, and more accessible. In addition to their low glycemic index just like foods currently used to manage diabetes, functional foods contain bioactive compounds such as polyphenols, dietary fibers, saponins, and peptides. They are so named because they have additional health advantages beyond their basic nutritional worth. Bioactive compounds can be found in a variety of SSA plant‐based foods, such as spices, fruits, vegetables, legumes, starchy foods, prepared foods, mixed foods, and prepared dishes. The goal of this review is to highlight some of the investigations into the effectiveness of local food and their antidiabetic mechanisms that have been studied in various SSA regions. Using the literature review as a basis, the authors state that SSA foods are rich in various bioactive compounds capable of regulating blood sugar through enhanced glucose tolerance, antioxidant effects, insulin sensitivity, and inhibition or activation of some key enzymes of the glucose metabolism that are linked to the prevention and management of T2DM. Many of the cited findings are preliminary, obtained from cell and preclinical studies, and therefore other studies need to be done to demonstrate the full potential of these foods to serve as bases for dietary guidelines.

## Graphical abstract

The major food groups consumed in SSA and especially the bioactive compounds found in them are listed in this review. The outcome demonstrates the richness of SSA foods in various compounds capable of regulating blood sugar by enhancing glucose tolerance, antioxidant, and insulin sensitivity, and inhibiting or activating some key enzymes of glucose metabolism that are useful to prevent and manage type 2 diabetes.

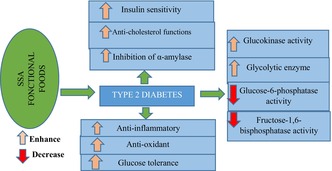

## 1 INTRODUCTION

The prevalence of diabetes was estimated at 9.3% in 2019 and was projected to increase to 10.9% by 2045. The urban population is the most affected by this growth. Indeed, 10.8% of this population is concerned, compared to 7.2% in rural areas (Saeedi et al., 2019). This ever‐increasing epidemiology concerns both developed and underdeveloped countries (Kotwas et al., 2021). Until 1985, diabetes was considered rare in SSA, with prevalence in many countries being less than 1% (Atun et al., 2017). Rapid urbanization, changes in lifestyle, dietary habits and the aging of the population are key factors, driving up the risk and prevalence of T2DM (Kotwas et al., 2021). Additionally, there is a high prevalence of other risk factors such as physical inactivity, obesity, and hypertension in the region (Juma et al., 2020). Furthermore, other factors, such as poverty, limited access to healthcare and a lack of awareness about the disease, also contribute to the high burden of diabetes in the region. Although the data are insufficient, the number of cases were estimated at 19.1 million in 2015 and it is projected to attain 41.4 million by the year 2035. This is an increase of 109% compared to 55% in the rest of the world (Diop & Diédhiou, 2015). Also, the International Diabetes Federation (IDF) projects that by 2045, there will be 47 million people in SSA with type 2 diabetes, up from 19.8 million in 2019 (IDF, 2019). The prevalence of diabetes varies across several countries in the region, with the highest prevalence rate in the Democratic Republic of the Congo, the United Republic of Tanzania, Nigeria, South Africa, and Ethiopia (Malek, 2021).

The increased burden of type 2 diabetes in sub‐Saharan Africa (SSA) represents a significant health challenge. This prevalence will continue to increase as many individuals with diabetes in SSA (60%) are not diagnosed thus, representing the highest percentage of non‐diagnosed diabetics from all the regions of the IDF (IDF, 2015). Almost three‐quarters of deaths from diabetes each year are related to people under 60 years.

Diet modification and increased physical activity are the first lines of treatment for T2DM. Nutritional care involves regular physical activity, alcohol and tobacco prohibition, etc., but above all, a diet adapted to the physiological state of the patient. It is important for individuals with diabetes to manage their blood glucose levels to prevent complications. The theoretical aspects of nutrition in the management of diabetes include understanding the role of carbohydrates, proteins, and fats in the body as well as the glycemic index, and the importance of portion control. Patients with diabetes are advised to eat a variety of fiber‐rich foods including, fruits, vegetables, legumes, and whole grains (ADA, 2019). They could eat foods rich in resistant starch or starch with a high percentage of amylose, all of which are poor in simple sugars and fats. Diabetic patients need to be especially careful about the kinds and amounts of carbohydrates they eat because high‐carbohydrate meals can quickly raise blood sugar levels. This is why understanding the glycemic index (GI) of foods is important. Low‐GI foods are recommended for people with diabetes because they have a positive effect on postprandial blood glucose levels (ADA, 2008). Fats are an essential part of the diet, but it is important to consume healthy fats, such as monounsaturated and polyunsaturated fats, and limit saturated and trans fats. Healthy fat consumption can lower cholesterol levels and lower the risk of heart disease, which is a frequent consequence cause of diabetes (Garonzi et al., 2021). Portion control is equally critical as ingesting the right proportions of carbohydrates, proteins, and fats is important in the control of blood glucose levels.

When nutritional therapy has failed, medicinal treatment is inevitable. Oral medications are generally used for regulating blood sugar and insulin secretion (Kotwas et al., 2021). In most countries of SSA, there is limited access to medication and monitoring equipment. Alternative therapy has been developed including the use of functional foods.

Functional foods are those that, in addition to their appropriate nutritional benefits, provide health effects that are curative, protective, or preventive of one or more diseases (Roberfroid, 2007). Although antinutrient substances (phytic acid, trypsin inhibitor, goitrogens, heavy metals, etc.) present in some of these foods reduce nutrient availability, these functional foods are beneficial to human health. For example, dietary fiber improves colon function and reduces plasma cholesterol levels; polyphenols and other substances have anticarcinogenic, antioxidants, anti‐inflammatory, antifungal, and many other preventive qualities (Martirosyan & Singh, 2015). Foods with bioactive substances, such as dietary fiber, polyphenols, saponins, and peptides, as well as those with a low glycemic index, are utilized to help diabetes patients maintain stable blood sugar levels. These substances, which are commonly present in plant‐based meals (fruits, vegetables, spices, grains, and legumes), regulate blood glucose levels through various mechanisms, including retardation of carbohydrate digestion and retardation of glucose absorption in the intestine, stimulation of insulin secretion from the pancreatic β‐cells, modulation of glucose release from the liver, activation of insulin receptors and glucose uptake in the insulin‐sensitive tissues, and modulation of glucose release from the liver (Kazeem & Davies, 2016).

SSA eat whole grain foods (millet, sorghum, and maize), which are rich in fiber; vegetables (okra, spinach, kale, and collard greens), which are high in fiber, minerals, vitamins, and low in calories; fruits (orange, papayas, guavas) which are high in fiber, vitamins, and antioxidant; and foods from animal sources which are rich in proteins and fats/oil (Oniang'o et al., 2003). These foods can also contain bioactive compounds that may be beneficial in managing diabetes. However, scientific evidence needs to be established to develop a personalized diabetes meal plan. Therefore, this review aims to explore articles about SSA food, their use in the management of diabetes, and information about their glycemic index.

## 2 AFRICAN DIET AND MANAGEMENT OF DIABETES

The African diet is diverse, and it varies by region, cultural beliefs, and socioeconomic status. However, traditional African diets are generally based on whole grains, vegetables, fruits, legumes, and lean protein sources. These diets are often low in saturated fat and high in fiber, contain phytochemical elements, and may be helpful in controlling diabetes and its consequences.

### 2.1 African herbs and spices

Indigenous spices and herbs are used in Africa to improve the flavor, taste, color, and shelf‐life of food. The physiological benefits of spices for health have been demonstrated in investigations on experimental animals as well as in human trials in recent decades, and they have been found to have a critical role in reducing DM complications (Srinivasan, 2005).

Herbs and spices have been thought of as potential antioxidant because of their reputation as great producers of phenolic chemicals. Due to their antioxidant properties, capacity to prevent the production of oxygen radicals in aerobic metabolism, and ability to interfere with signal transduction pathways, spices like clove containing eugenol, turmeric containing curcumin, and red pepper containing capsaicin have been experimentally demonstrated to control oxidative stress in cells (Scalbert & Williamson, 2000). Due to the bioactive compounds found in spices, they also have remarkable potential for medicinal use, including hypoglycemic effects (Mohammed & Islam, 2018). Pancreatic α‐amylase can be inhibited by an ethanolic preparation of the African nutmeg, *M. myristica* seed. This enzyme helps convert starch and glycogen into glucose. Its inhibition is a useful method for controlling blood glucose by reducing glucose release and absorption (Okonji et al., 2015). In vitro inhibition of α‐amylase and α‐glucosidase was demonstrated by the essential oils from the seeds of *A. melegueta* and *A. danielli*. This antihyperglycemic effect is thought to be caused by the combination of eugenol, limonene, α‐pinene, and β‐pinene in these oils (Adefegha et al., 2017). In diabetic rats, *A. melegueta* methanolic extract has also been shown to control hyperglycemia (Elizabeth et al., 2019). Non‐volatile phytochemicals such as paradols, shogaols, and gingerols are present in ginger (Ali et al., 2008). Among these substances, 6‐shogaol (5 or 10 mg/kg body weight) has demonstrated an antidiabetic effect by significantly lowering blood sugar and body weight levels, attenuating the aforementioned pathological changes to normal levels in diabetic mice, and protecting their pancreas, kidneys, and liver from damage (Yi et al., 2019). In mice with alloxan‐induced diabetes, piperine, a key alkaloid present in the Piper genus, at dose of 20 mg/kg restores normal blood glucose levels (Atal et al., 2012). From *X. aethiopica* fruit, active antidiabetic compounds such as oleanolic, kaurenoic, and xylopic acids were identified. While kaurenoic and xylopic acids showed glucose‐lowering action, oleanolic acid reduced the activities of α‐glycosidase and α‐amylase (Famuyiwa et al., 2018; Mohammed et al., 2021). The antidiabetic properties of *Ocimum gratissimum* are due to the abundance of bioactive substances such as L‐chicoric acid, eugenyl‐d‐glucopyranoside, linolenic acid, l‐caftaric acid, and vicenin‐2 (Casanova et al., 2014).

### 2.2 African fruits and vegetables

Fruits are a great source of fiber, minerals, and vitamins, all of which are necessary for a balanced diet. Table 1 shows some traditional African fruits and vegetables containing high levels of iron, folic acid, vitamin C, and β‐carotene. *C. olitorius*, *A. digitata* leaf, and *A. cruentus* are comparable in folic acid content as cabbage (66 μg/100 g), broccoli (105 μg/100 g), and spinach (193 μg/100 g) (Aworh, 2014).

**TABLE 1:** Traditional African fruits and vegetables with high contents in vitamin C, β‐carotene, folic acid, and iron.

Vitamin C (mg/100 g) (Charles Aworh, 2015; Stadlmayr et al., 2013)		β‐carotene (μg/100 g) (Stadlmayr et al., 2012)		Folic acid (μg/100 g) (Stadlmayr et al., 2012)		Iron (mg/100 g) (Charles Aworh, 2015)	
Baobab (*Adansonia digitata*) fruit pulp	300	*Corchorus Olitorius*	3130	*C. Olitorius*	118	*Solanum aethiopicum*	18
*Thuja occidentalis*	129	*A. cruentus*	2890	*A. digitata* leaf	118	*C. argentea*	13
*C. olitorius*	78	*Hibiscus sabdariffa* leaf	2580	*A. cruentus*	79	*T. occidentalis*	10
*Gnetum africanum*	56					*A. digitata* fruit pulp	9
*A. cruentus*	56					*A. cruentus*	6
*Irvingia gabonensis var. gabonensis*	54					*C. olitorius*	6
*C. albidum*	48						
*A. digitata* leaf	47						

The nutritional value of traditional green vegetables in KwaZulu‐Natal, South Africa, reveals that 12 of them, including *Amaramthus dubius*, *Asystasia gangetica*, *Amaranthus hybridus*, *Amaranthus spinosus*, *Cncumis metlllijerus*, *Cleome monophylla*, *Ceratotheca tribola*, *Galinsoga parvi‐flora*, *Jusuca flava*, *Momordica balsamina*, *Physalis viscosa*, and *Wohlcnbergia undulata*, had mineral concentrations above 1% of plant dry weight, which were significantly higher (Odhav et al., 2007).

Low‐GI fruits including cherries, prunes, grapefruit, dried apricots, raisins, peaches, apples, pears, strawberries, plums, guava, oranges, grapes, papaya, banana, kiwi, pineapple, figs, and mango can still be consumed by diabetics (Asif, 2011). A lower risk of diabetes has been linked to eating vegetables. In this instance, onions can provide up to 20% of the daily need for chromium (Dias, 2012). By boosting in vivo insulin activity and potentially activating insulin receptor kinases, chromium increases glucose tolerance (Wang et al., 2005). Clinical investigations in diabetic patients have demonstrated that chromium is able to decrease total cholesterol and triglyceride levels, enhance glucose tolerance, lower insulin levels, and lower fasting blood glucose and insulin levels (Dias, 2012).

Many phytochemicals in fruits that have antioxidant properties can protect β‐cells from oxidative damage. It is the case with citrus fruits that contain active compounds like flavanones (neohesperidin, narirutin, naringin, naringenin, hesperidin, eriocitrin, and didymin), flavones (5‐hydroxy‐3,6,7,8,3′,4′‐hexamethoxyflavone, 5‐demethylnobiletin, 3′,4′,3,5,6,7,8 heptamethoxyflavone, tangeretin, sudachitin, sinensetin, nobiletin, diosmin, and diosmetin) and others flavonoids (anthocyanins, cigranoside C, D, E, F, and quercetin) (Lu & Yip, 2023). Didymin substantially inhibited advanced glycation end products, rat lens aldose reductase, human recombinant aldose reductase, protein tyrosine phosphatase 1B, and α‐glucosidase. Additionally, by activating the PI3K/Akt signaling pathway in insulin‐resistant HepG2 cells, it boosted glucose absorption in those cells (Ali et al., 2019). Type 2 diabetic rats that had been treated with nicotinamide/streptozotocin, hesperidin, and quercetin at a dose of 100 mg/kg body weight/day showed increased insulin secretion, insulin action, and the antioxidant defense system (Ali et al., 2020). Gluconeogenesis, redox imbalance, and cholinergic dysfunction are all simultaneously suppressed by the African star apple (*Chrysophyllum albidum*), while muscular shape is preserved and elemental dysfunction is moderated (Ajayi et al., 2020). Oranges, grapefruit, and lemons are sources of the monocyclic monoterpene d‐limonene (p‐menthal‐1, 8‐diene) (Murali & Saravanan, 2012). The antidiabetic effect of d‐limonene (50, 100, and 200 mg/kg body weight) has been proven in streptozotocin‐induced diabetic rats by a decrease in blood sugar and glycosylated hemoglobin levels, a decrease in the activities of fructose‐1,6‐bisphosphatase and glucose‐6‐phosphatase, and an increase in the activity of the glycolytic enzymes and glucokinase (Murali & Saravanan, 2012). Tomatoes (*Lycopersicum Esculentum*) work by controlling blood sugar levels in diabetics. It contains the pigment lycopene, which lowers blood sugar levels by lowering insulin resistance (Tarigan et al., 2020). Additionally, tomatoes contain kaempferol, a dietary flavonoid that boosts antioxidant capacity and lowers indicators of lipid peroxidation (Al‐Numair et al., 2015). Due to its high concentration of lycopene and β‐carotene, tomato juice has been found to have hypoglycemic and antiatherogenic benefits in diabetic rats (Khalil et al., 2022). According to Esmaeilzadeh et al. (2020), quercetin, fiber, and polysaccharides like rhamnoglacturonan present in okra have antidiabetic potentials. They can act through a variety of mechanisms, including antioxidants, a reduction in glucose absorption from the intestinal tract, an inhibition of DPP‐4 activity, a downregulation of pancreatic PPARs, an inhibition of α‐glucosidase, and a reduction of liver insulin degradation enzyme and TNF‐α gene expression (Esmaeilzadeh et al., 2020).

### 2.3 African starchy food

Subterranean stems, roots, rhizomes, corms, and tubers that are starchy store edible starch content. In diabetic rats, cocoyam corm, a starchy meal, has been proven to lower postprandial blood glucose levels (Oluyemisi et al., 2016). Hypoglycemic actions of unripe plantain in diabetic animals have been reported (Eleazu & Okafor, 2015). The flavonoids, stilbenes, tannins, and saponins found in edible cassava leaves are capable of inhibiting the activities of α‐amylase, α‐glucosidase, and lipase (Laya et al., 2022). Mucin, dioscin, dioscorin, aJlantoin, choline, polyphenols, diosgenin, carotenoids, tocopherols, and water‐soluble polysaccharides (WSP) are only a few of the bioactive substances found in yam (Estiasih et al., 2012; Muyonga et al., 2018). In hyperglycemia, yam WSP extracts had the potential to lower blood sugar levels while also preventing SCFA production and glucose absorption (Estiasih et al., 2012). The amount of phytochemicals in sweet potato roots varies depending on the type and flesh color, including carotenoids, tocopherols, phenolic compounds, tannins, flavonoids, and saponins (Mohanraj & Sivasankar, 2014). According to Matsui et al. (2004), acylated anthocyanins like caffeoyl sophorose, which serves as an α‐glucosidase inhibitor, are what give sweet potatoes their antidiabetic properties. In a study involving rats, white‐skinned sweet potato (WSSP; *Ipomoea batatas* L.) peels (4 g/kg/day) demonstrated the ability to reduce blood sugar, protein glycation, total cholesterol, triglycerides, and low‐density lipoprotein LDL cholesterol. There was also a reported rise in HDL cholesterol, a type of high‐density lipoprotein (Akhtar et al., 2018). Sweet potatoes contain phenolic compounds, dietary fiber, and resistant starch, which have been shown to have antidiabetic effects by lowering fasting blood sugar and insulin resistance. In contrast, phenolic compounds, anthocyanins, carotenoids, tocopherols, flavonoids, and ascorbic acid have antioxidant effects (Akhtar et al., 2018; Ooko Abong' et al., 2020).

Millet and sorghum are important crops for populations in Africa, they are rich in bioactive ingredients (Table 2). In both healthy people and people with type‐2 diabetes, millet has a favorable influence on fasting and postprandial blood glucose levels as well as the plasma–insulin response (Almaski et al., 2019). In STZ‐induced diabetic mice, sorghum extract (0.4 g/kg body weight) lowers blood glucose levels through suppression of hepatic gluconeogenesis by reducing PEPCK and p38 expression and increasing AMPK expression (Kim & Park, 2012).

**TABLE 2:** Bioactive compounds of millet and sorghum (Vila‐Real et al., 2017).

Components	Finger millet	Sorghum
Ferulic (μg/g)	186	120.5–173.5
Protocatechuic acid (μg/g)	450	150.3–178.2
Caffeic acids (μg/g)	16.4	13.6–20.8
Tannins (μg/g)	/	0.2–48.0
Flavonoids (μg/g)	/	87
Total phenol (mg/100 g)	102	Phenolic acids: 135.5–479.40 μg/g

### 2.4 Formulated food, mixed food, and prepared dishes

Formulated and mixed foods are necessary because we expect that they improve the antidiabetic effects and exclude the monotony linked to a particular diet. Cocoyam and unripe plantain combination showed antihyperglycemic and antihyperlipidemic action but these effects are less important than Cocoyam or unripe plantain alone (Eleazu et al., 2016). In streptozotocin‐induced diabetic rats, the combination of ginger and unripe plantains has also demonstrated antihyperglycemic effects. However, in comparison to unripe plantains alone, the findings were not conclusive (Iroaganachi & Eleazu, 2015). Patients with type II diabetes mellitus can lower their blood glucose levels by drinking tomato and guava juice together (Hasneli & Mardhiyah, 2019).

Mbanya et al. (2003) evaluated the glycemic index, insulinemia index, and in vitro digestibility of the five most popular mixed meals in Cameroon, which include rice and tomato soup, bean stew and plantains, corn fufu and ndole, yams and groundnut soup, koki beans, and cassava. The findings demonstrate the significance of eating mixed meals because the GI is much lower in mixed meals than it is in single servings of carbohydrate‐rich foods (Mbanya et al., 2003). Garri and afang soup, pounded yam, and edikang ekong soup consumption significantly lowered fasting blood glucose levels (Ani et al., 2011).

Table 3 below summarize the antidiabetic effect of some SSA diet.

**TABLE 3:** Sub‐Saharan African foods with antidiabetic effect.

Foods type	Mode of action	Bioactive compounds	References
Yellow soup with sissongo leaves	Lower increase in the plasma glucose concentration		Christine Fernande et al. (2022)
African star apple	Inhibit α‐glucosidase and α‐amylase activities and exhibit antioxidant properties	β‐Amyrin acetate, eleagine, epicatechin, epigallocatechin, skatole, stigmasterol, and tetrahydro‐2‐ methylharman	Ajayi et al. (2020)
Tomatoes	Decrease blood sugar levels	Lycopene and chromium	Tarigan et al. (2020)
Millet	Beneficial effect on fasting and postprandial blood glucose and the plasma–insulin response	Polyphenols and antioxidants	Almaski et al. (2019)
Ginger	Significantly decreasing the level of blood glucose and body weight. He has an effect against pancreas, kidney, and liver damage in the diabetic mice	6‐gingerol	Yi et al. (2019)
White‐skinned sweet potato	Decreased blood glucose level and lowered insulin resistance	Caffeoyl sophorose, phenolic compounds, dietary fiber, and resistant starch	Akhtar et al. (2018)
Cocoyam and unripe plantain combination	Antihyperglycemic and antihyperlipidemic action	Saponins, flavonoids, tannins, and alkaloids	Eleazu et al. (2016)
Cocoyam corm	Reducing the postprandial blood glucose level of alloxan‐induced diabetic rats	Phenolic and flavonoid compounds	Oluyemisi et al. (2016)
Unripe plantain flour	Hypoglycemic actions and amelioration of acute pancreatitis	Saponins, flavonoids, tannins, and alkaloids	Eleazu and Okafor (2015)
Ethanolic extract of *M. myristica* seed	Ability to inhibit pancreatic α‐amylase	Phenolic compounds	Okonji et al. (2015)
Rice with groundnut sauce	Lower increase of the plasma glucose concentration, as a result of slower rates of gastric emptying and digestion of carbohydrates in the intestinal lumen		Kouamé et al. (2014)
Cassava starch	Decreased the plasma total cholesterol level and LDL cholesterol level, and improved the plasma HDL cholesterol level and intestine short‐chain fatty acid content	Resistant starch	Wang et al. (2014)
Local Kokonte	Lower increase in the plasma glucose concentration		Akinlua (2013)
Onions	Decrease fasting glucose levels, improve glucose tolerance, and lower insulin levels	Chromium	Dias (2012)
Yam	Decrease blood glucose levels in hyperglycemia conditions as well as inhibited glucose absorption and SCFA formation	Dioscorin and water‐soluble polysaccharides	Estiasih et al. (2012)
Sorghum flour	Reduces blood glucose concentration in STZ‐induced diabetic rats by inhibiting hepatic gluconeogenesis through suppression of PEPCK and p38 expression and increases AMPK expression.	Phytochemicals, tannins, phenolic acids, anthocyanins, phytosterols, and policosanols	Kim and Park (2012)
Essential oil from *A. melegueta* seed	In vitro inhibition of α‐amylase and α‐glucosidase	Eugenol, limonene, α‐pinene, and β‐pinene	Murali and Saravanan (2012)
Orange, grapefruit, and lemon	Decrease plasma glucose, glycosylated hemoglobin levels, activities of gluconeogenic enzymes such as, glucose‐6‐phosphatase and fructose‐1,6‐bisphosphatase, and an increase in the glycolytic enzyme and glucokinase activity	D‐limonene (p‐menthal‐1, 8‐diene)	Murali and Saravanan (2012)
Garri with afang soup	Reduced fasting blood glucose results were significantly		Ani et al. (2011)
Okra	Reduced postprandial blood glucose by reducing the diffusion of glucose and postponing the absorption and digestion of carbohydrates	Viscous water‐soluble dietary fibers	Khatun et al. (2011)

## 3 GLYCEMIC INDEX OF SOME SSA FOOD

As previously said, diabetic patients should eat foods with a low GI (Table 4). Research on regional foods, such as that conducted by Kouassi et al. (2009), has revealed that the GI values of yams often consumed in Côte d'Ivoire vary across a sizable range from 51 to 70 (Kouassi et al., 2009). According to Kouamé et al. (2014), while rice with groundnut sauce has a low GI (GI = 45), foods like pounded yam with eggplant sauce and cassava paste with granulated palm nut sauce, which are both consumed in Côte d'Ivoire, have high GIs ranging from 94 to 86, respectively (Kouamé et al., 2014). In a study conducted by Serwaa Yeboah et al. (2019), who looked into how processing affected the glycemic index of five Ghanaian corn and cassava staples, locally produced kokonte had the lowest GI of 7, followed by processed kokonte, which had a GI of 18, and low‐GI kafa, which had a GI of 29. Abolo and Akple both had medium glycemic index values of 58 and 69, respectively (Serwaa Yeboah et al., 2019). Based on Akinlua (2013) study on the GI of selected Nigerian foods, beans served with stew had a GI of 56, akara had a GI of 44, moin‐moin had a GI of 41, and ofuloju had a GI of 54 (Akinlua, 2013). When fufu corn is combined with certain sauces, such as nkui, it lowers its glycemic index significantly, making it an excellent candidate for inclusion in diet plans for those with diabetes and other metabolic diseases (Kamwa et al., 2015).

**TABLE 4:** Sub‐Saharan African foods with low glycemic index.

Tested diet	GI	Reference food	CHP / serving size (g)	Area of study	References
Tchoukoutou	20.59	Bread	50/100	Benin	Gomina et al. (2016)
Sodabi	18.34	Bread	50/100	Benin	Gomina et al. (2016)
Fermented palm wine	4.27	Bread	50/100	Benin	Gomina et al. (2016)
Fresh palm wine	11.66	Bread	50/100	Benin	Gomina et al. (2016)
Bean stew + plantains	48.36	Glucose	75	Cameroon	Mbanya et al. (2003)
Black sauce and okra	8.33	White bread	/200	Cameroon	Christine Fernande et al. (2022)
Foofoo corn + ndole	34.06	Glucose	75	Cameroon	Mbanya et al. (2003)
Rice + tomato soup	46.33	Glucose	75	Cameroon	Mbanya et al. (2003)
Yams + groundnut soup	49.51	Glucose	75	Cameroon	Mbanya et al. (2003)
Banana cake with chili leaves	27.27	White bread	/200	Cameroun	Christine Fernande et al. (2022)
Baobab soup–beef meat/fufu corn	25.0	White bread	/200	Cameroun	Christine Fernande et al. (2022)
Baobab soup–beef meat/fufu rice	33.33	White bread	/200	Cameroun	Christine Fernande et al. (2022)
Crush irish potatoes with dried yams	36.36	White bread	/200	Cameroun	Christine Fernande et al. (2022)
Dried lalo–beef meat/fufu corn	16.67	White bread	/200	Cameroun	Christine Fernande et al. (2022)
Dried lalo–beef meat/Fufu rice	16.67	White bread	/200	Cameroun	Christine Fernande et al. (2022)
Dried okra–beef meat	2.08	White bread	/200	Cameroun	Christine Fernande et al. (2022)
Eggplants puree	33.33	White bread	/200	Cameroun	Christine Fernande et al. (2022)
Folere–beef meat/fufu rice	16.67	White bread	/200	Cameroun	Christine Fernande et al. (2022)
Fresh lalo–beef meat/fufu rice	4.17	White bread	/200	Cameroun	Christine Fernande et al. (2022)
Fresh okra–beef meat/fufu cassava	16.67	White bread	/200	Cameroun	Christine Fernande et al. (2022)
Fresh peanut with huckleberry	18.18	White bread	/200	Cameroun	Christine Fernande et al. (2022)
Green soup	25.00	White bread	/200	Cameroun	Christine Fernande et al. (2022)
Huckleberry leaves	1.51	White bread	/200	Cameroun	Christine Fernande et al. (2022)
Kneaded yams	21.21	White bread	/200	Cameroun	Christine Fernande et al. (2022)
Koki dried plantain	45.83	White bread	/200	Cameroun	Christine Fernande et al. (2022)
Koki macabo	4.17	White bread	/200	Cameroun	Christine Fernande et al. (2022)
Melon leaves	20.83	White bread	/200	Cameroun	Christine Fernande et al. (2022)
Melon with eggplants	12.12	White bread	/200	Cameroun	Christine Fernande et al. (2022)
Paï leaves	29.17	White bread	/200	Cameroun	Christine Fernande et al. (2022)
Pounded macabo with roasted vegetables	33.33	White bread	/200	Cameroun	Christine Fernande et al. (2022)
Roasted caterpillar	24.24	White bread	/200	Cameroun	Christine Fernande et al. (2022)
Saka'a leaves	33.33	White bread	/200	Cameroun	Christine Fernande et al. (2022)
Soye/fufu cassava	25.0	White bread	/200	Cameroun	Christine Fernande et al. (2022)
Termites dishes	20.83	White bread	/200	Cameroun	Christine Fernande et al. (2022)
Yellow sauce and eggplants	4.17	White bread	/200	Cameroun	Christine Fernande et al. (2022)
Yellow sauce and elephant grass	33.33	White bread	/200	Cameroun	Christine Fernande et al. (2022)
Yellow sauce and okra	45.83	White bread	/200	Cameroun	Christine Fernande et al. (2022)
Yellow soup with sissongo leaves	3.03	White bread	/200	Cameroun	Christine Fernande et al. (2022)
Young cocoa fruits sauce	1.21	White bread	/200	Cameroun	Christine Fernande et al. (2022)
Young sissongo leave	39.39	White bread	/200	Cameroun	Christine Fernande et al. (2022)
Young sissongo sprouts	16.67	White bread	/200	Cameroun	Christine Fernande et al. (2022)
Rice with groundnut sauce	45	Glucose	50	Côte d'Ivoire	Kouamé et al. (2014)
Teff Injera	35.6	Glucose	50/20.2	Ethiopia	Dereje et al. (2019)
Anchovies	0.9	Glucose	50	Ghana	Serwaa Yeboah et al. (2019)
Groundnut soup with beef	4	Glucose	50	Ghana	Serwaa Yeboah et al. (2019)
Whole maize *ugali*, cowpea leaves	45	Glucose	50/158	Kenya	Ebere et al. (2021)
Akara	43.70	Glucose	/180	Nigeria	Akinlua (2013)
Cassava flour	40.12	Glucose	50	Nigeria	Fasanmade and Anyakudo (2007)
Maize flour	26.61	Glucose	50	Nigeria	Fasanmade and Anyakudo (2007)
Moin‐moin	41.14	Glucose	/180	Nigeria	Akinlua (2013)
Yam flour	35.30	Glucose	50	Nigeria	Fasanmade and Anyakudo (2007)

Abbreviations: CHP, carbohydrate portion; GI, glycemic index.

Christine Fernande et al. (2022) have determined the glycemic index of 41 Cameroonian local meals usually consumed and cooked. Among them, some meals like yellow sauce and eggplants, lalo‐beef meat with fufu rice, yellow soup with sissongo leaves, young cocoa fruit sauce, huckleberry leaves, koki macabo, and dried okra–beef meat have a GI lower than 5 (Christine Fernande et al., 2022).

## 4 CONCLUSIONS AND FUTURE PROSPECTS

SSA continues to see growth in the number of people with type 2 diabetes despite research improvements. One of the strategies to fight against this alarming progression would be to carry out local research work on foods for daily consumption to highlight not only their nutritional role but also their potential functional effect. The findings of the present review enable us to draw the conclusion that SSA food has hypoglycemic effect that can be affected by food composition, glycemic index, processing techniques (cooking, grinding, fermentation, etc.), and bioactive compounds present in them. The antioxidant and antidiabetic qualities of dishes can be enhanced by adding herbs and spices in the preparation. African starchy food contains more bioactive compounds whose antioxidants and antidiabetic activities have been reported. They can be used to formulate food, mix food, and prepare dishes. To give some detailed explanation regarding this aspect, further recommended studies are therefore necessary. Among other things, Some African countries still do not have data on the glycemic index of their local foods, and this can represent an obstacle in the management of diabetes. Indeed, knowing fully well the importance of a dietary plan for people living with diabetes, each African country needs to list its local functional foods capable of managing it. This list will allow medical doctor to be able to prescribe a diet adapted to local realities.

## AUTHOR CONTRIBUTIONS

**Rosane Matsinkou Soh:** Conceptualization (lead); data curation (lead); formal analysis (lead); investigation (lead); methodology (equal); resources (lead); supervision (equal); writing – original draft (lead); writing – review and editing (lead). **Wilfred Damndja Ngaha:** Investigation (supporting); methodology (equal); supervision (equal); writing – original draft (supporting); writing – review and editing (supporting). **Nicolas Njintang Yanou:** Methodology (supporting); supervision (equal); validation (supporting); visualization (supporting); writing – original draft (supporting); writing – review and editing (supporting).

## CONFLICT OF INTEREST STATEMENT

The authors declare no conflicts of interest.

## ETHICS STATEMENT

Not applicable.

## Data Availability

Data sharing is not applicable to this article as no datasets were generated or analyzed during the current study.
